# Visual processing is diminished during movement execution

**DOI:** 10.1371/journal.pone.0213790

**Published:** 2019-03-21

**Authors:** Joëlle Hajj, Dana Maslovat, Erin K. Cressman, Laura St. Germain, Anthony N. Carlsen

**Affiliations:** 1 School of Human Kinetics, University of Ottawa, Ottawa, Canada; 2 School of Kinesiology, University of British Columbia, Vancouver, Canada; University of Wuerzburg, GERMANY

## Abstract

Recent research has suggested that visual discrimination and detection may be enhanced during movement preparation and execution, respectively. The current study examined if visual perceptual processing is augmented prior to or during a movement through the use of an Inspection Time (IT) task. The IT task involved briefly presenting (e.g., 15–105 ms) a “pi” figure with differing leg lengths, which was then immediately masked for 400 ms to prevent retinal afterimages. Participants were subsequently required to choose which of the two legs was longer. In Experiment 1, participants (n = 28) completed the IT task under three movement conditions: no-movement (NM), foreperiod (FP), and peak velocity (PV). In the NM condition, participants solely engaged in the IT paradigm. In the FP condition, the IT stimulus was presented prior to movement execution when response planning was expected to occur. Finally, in the PV condition, participants made a rapid movement to a target, and the IT stimulus was presented when their limb reached peak velocity. In Experiment 2, participants (n = 18) also performed the IT task in the PV and NM condition; however, vision of the limb’s motion was made available during the PV trials (PV-FV) to investigate the potential influence of visual feedback on IT performance. Results showed no significant differences in performance in the IT task between the NM and FP conditions, suggesting no enhancement of visual processing occurred due to response preparation (Experiment 1). However, IT performance was significantly poorer in the PV condition in comparison to both the NM and FP conditions (Experiment 1), and was even worse when visual feedback was provided (Experiment 2). Together, these findings suggest that visual perceptual processing is degraded during execution of a fast, goal-directed movement.

## Introduction

In their seminal work, Goodale and Milner [1] showed that vision for perception is processed differently and via a separate pathway compared to vision for action. In particular, Goodale and Milner’s dual systems model suggests that visual information used to guide movements online is processed by a dorsal visual stream, whereas visual perceptual processing occurs predominantly via a much slower ventral visual stream. Although perceptual processing is the slower of the two modes, some anecdotal accounts have suggested that visual perceptual processing may be upregulated during movement. For example, professional baseball and tennis players have reported experiencing a “slowing-down” of the ball just prior to contact. Recent studies have investigated whether this purported upregulation of visual perception might occur before [2] and/or during [3] goal-directed movement. For example, Hagura and colleagues [2] investigated whether planning for a goal-directed movement alters visuo-temporal discrimination ability. They found that motor preparation not only influenced the perception of the duration of a visual stimulus, but also the perceived rate of flow of visual information, as rapidly presented sequences of letters were more accurately perceived when presented just prior to a reaching movement [2].

Other research has shown that detection of visual stimuli may be enhanced when presented during a goal-directed movement at high limb velocities, in comparison to lower limb velocities [3]. Specifically, Tremblay and Nguyen [3] demonstrated that participants’ susceptibility to the “fusion illusion,” where two visual stimuli (e.g., two flashes) are perceived as a single visual stimulus when they are accompanied by a single acoustic stimulus [4], was altered depending on when it was presented during a goal directed movement. Specifically, the incidence of incorrectly reporting the number of flashes (i.e., susceptibility to the fusion illusion) was significantly decreased when it was presented at peak-limb velocity during a manual pointing task, compared to when it was presented at lower limb velocities. Although an unpublished study by Goodman and Tremblay [5] found that participants were less likely to report the correct number of flashes in a visual flash detection task without a concurrent acoustic stimulus when the limb was moving at peak-limb velocity (in comparison to at all other movement velocities), a follow-up study confirmed a diminished suceptibility to the audio-visual fusion illusion at peak-limb velocity, but also included a condition where there was no movement [6].These results suggested that either 1) the speed of visual perceptual processing was enhanced when visual information was important for limb regulation, or 2) multi-modal stimulus processing was improved during limb movement [3].

In order to determine whether visual processing ability is indeed altered by movement preparation or execution, it is critical to use a task that is assumed to act as a measure of visual processing speed. Inspection time (IT) is a psychophysical paradigm that measures the amount of time that a visual stimulus must be presented in order for it to be accurately discriminated by a participant [7]. The visual stimulus most commonly used is a “pi” figure with differing leg lengths which is briefly presented (e.g., 20–200 ms) and then rapidly backward masked to prevent further visual processing. Participants are typically required to indicate which side (left or right) of the pi figure has the longest leg. When the pi figure is presented for a brief amount of time (e.g., 20 ms), response accuracy does not differ from chance (~50%). However, as the pi figure is presented for longer periods of time, participants are more likely to correctly determine which leg of the pi figure was longest, with nearly 100% accuracy achieved at longer presentation times (e.g., 200 ms). Performance accuracy on the IT task can be directly linked to perceptual visual processing speed and as such, avoids participants having to integrate multiple sources of sensory information as in Tremblay and Nguyen’s [3] study. As well, the IT task avoids any potential practice/familiarity effects that may be associated with other types of frequently encountered visual stimuli (e.g., letters of the alphabet).

Here we report two experiments in which the IT task was presented prior to, or during the performance of a goal-directed movement. In Experiment 1, the IT task was performed either in a no-movement condition, at peak velocity of the movement, or during the foreperiod (prior to movement onset). The no-movement condition served as a baseline to assess IT performance when participants remained idle. The peak velocity condition was employed to investigate whether visual processing (i.e., IT performance) would be enhanced during movement execution. Finally, the foreperiod condition assessed whether preparing to perform a goal-directed movement would affect IT performance. In Experiment 2, participants received visual feedback regarding their hand position during the PV condition (PV-FV), to establish if the availability of online visual feedback influenced perceptual visual processing. In addition to reporting which leg was the longest in the IT task, participants were required to indicate their confidence in their report. These subjective confidence rating scores (on a scale of 1 to 5) were added in order to increase participant engagement in the IT task and capture their perceptual experience (i.e., their level of conscious awareness).

The purpose of Experiment 1 was to determine whether visual processing ability, as indexed by IT performance would be altered by planning (foreperiod condition) or executing a goal-directed movement (peak velocity condition) in a reaction time (RT) paradigm compared to a no-movement (baseline) condition. Based on the work by Hagura et al. [2], we hypothesized that visual IT would be shorter (i.e., participants would be more accurate in identifying the longest leg of the figure at various display durations) when the pi-stimulus was presented during the RT foreperiod compared to trials in which no movement was required. Moreover, we hypothesized that if perceptual processing was enhanced when the limb was moving at peak-limb velocity, then visual IT would be shorter in comparison to trials in which no movement was required. The purpose of Experiment 2 was to investigate the influence of online visual feedback on IT task performance during a goal-directed movement. It was hypothesized that the provision of online visual feedback would lead to greater usage of the visual system, which would in turn lead to a reduced amount of time needed to perceive the IT stimulus. In contrast to our hypothesis, however, visual IT was found to be longer at peak-limb velocity in comparison to the no-movement and foreperiod conditions in Experiment 1, and was even longer at peak-limb velocity when full vision was available in Experiment 2.

## Experiment 1

### Material and methods

#### Participants

Twenty-eight healthy individuals (15 F, 13 M), with a mean age of 25.9 years (SD = 4.8) participated in Experiment 1. All participants self-reported as right-handed or ambidextrous. Testing took place in a single session at the University of Ottawa. All participants provided written informed consent prior to testing. This study was approved by the University of Ottawa Research Ethics Board (REB approval: H04-16-01) and conformed to the latest revision of the Declaration of Helsinki.

#### Set-up

**Fig 1:**
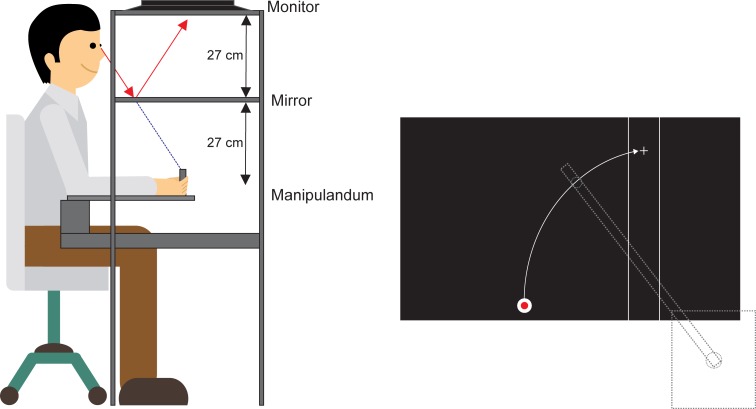
Schematic depiction of the experimental apparatus and display. The left part of the figure depicts a participant and the experimental apparatus with computer screen mounted facing downwards and the participant seeing the reflected display. Participants gripped the vertical handle of the manipulandum situated under the mirror. The right side shows a depiction of how the manipulandum handle travelled in all movement conditions during the 30^o^ elbow extension from the home position (white circle) to the target region (between the vertical lines). The grey dashed object represents the manipulandum arm, situated under the mirror and thus hidden from view.

Participants sat in a height adjustable chair whose height and distance from a table was adjusted to ensure that participants could comfortably reach to targets and view visual stimuli. The participant’s right arm was placed in a custom-made manipulandum with a vertical handle on the end that allowed for arm flexion/extension movements about the elbow such that the handle could move in a fixed arc in the horizontal plane towards and away from the body. At the start of each trial, the arm was at the “home” position, such that the shoulder was flexed approximately 45 deg, abducted 15 deg, and internally rotated 15 deg, with the elbow flexed at 90 deg. Thus, the medial aspect of the forearm was pointed downward and parallel to the floor and the hand gripped the handle comfortably. Visual stimuli were displayed on an ASUS VG248QE LCD monitor (refresh rate of 144 Hz and a response time of 1 ms) which was mounted facing downwards 27 cm above a reflective mirror. The manipulandum was located 27 cm below the reflective mirror, such that the participant’s hand was hidden from view and the stimuli appeared to be in the same plane as the manipulandum (see Fig 1 for visual depiction of participant position and visual display). A linear potentiometer attached to the pivot point of the manipulandum was used to measure elbow angular displacement (deg), which was then used to calculate angular velocity (deg/s) in real time (~5 ms lag).

#### Stimuli

Visual stimuli were presented on the monitor and viewed as a reflected image on the mirrored surface. The home position was located approximately 20 cm directly in front of the participant in line with their midline and consisted of a white dot, 1 cm in diameter presented against a black background. The target region consisted of two white vertical lines (3 cm apart and extending the vertical length of the screen) presented against a black background approximately 15 cm to the right of the midline. The home position and target region were always visible. At the start of each trial a fixation cross (1 cm x 1cm) was presented centrally inside the target region (7.5 cm from the top of the screen, and approximately 40 cm in front of the participant), which corresponded to a clockwise angular rotation from the home position of 30 deg (see Fig 1). The distance from the home position to the center of the target region along the curvilinear path was 16.25 cm. The fixation cross was removed and after 300 ms a white “pi” figure was presented inside the target in the location of the fixation cross, with the legs of the pi figure differing in length. One leg of the pi figure was 5.7 cm long while the other was 7 cm long. Both legs were 0.3 cm inside the edges of the target region and joined at the top by a 2.4 cm horizontal line, 5 cm from the top of the screen. In a random order, the longest leg equally appeared on the right and left side of the figure. In order to limit processing of the stimulus, the pi figure was backward masked for 400 ms by two 8.1 cm long legs which were composed of triangular shaped protrusions centered at 5.7 cm from the top of the figure (e.g., lightning mask; for a graphical depiction of similar stimuli see [8]). Visual feedback of limb position was provided by a 0.5 cm diameter red dot that indicated the position of the handle of the manipulandum. Feedback of limb position was given at the end of each trial to provide terminal feedback and remained available until the start of each trial to allow the participant to return to the home position (see below).

### Procedure

**Fig 2:**
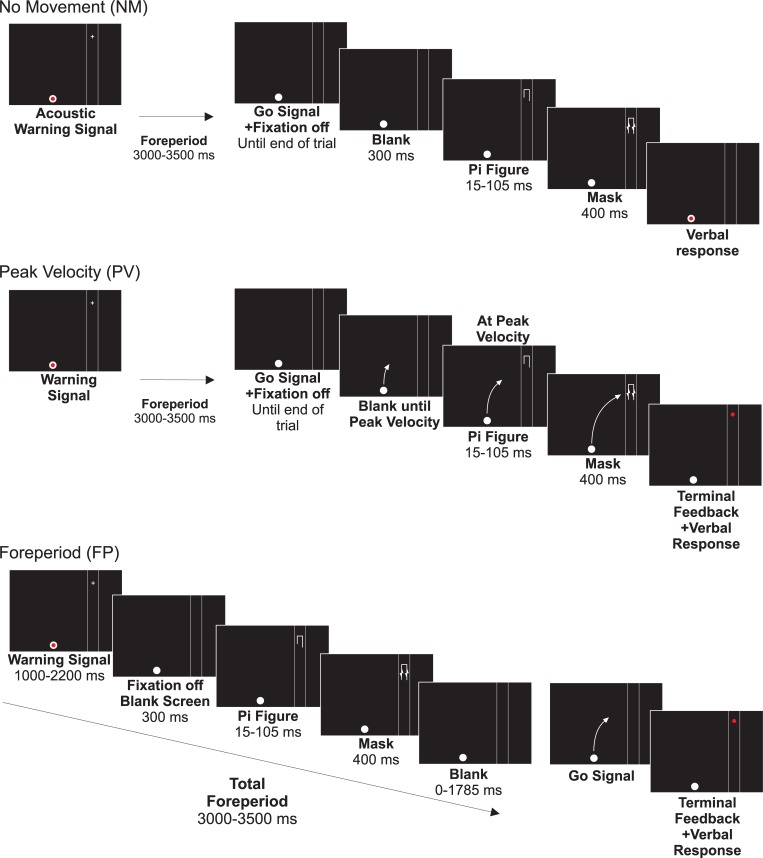
A chronological depiction of the visual stimuli presented in the NM, PV, and FP conditions. For each condition, a representation of the duration of stimuli presented and feedback received by participants at various times is provided. Note that the curved arrows schematically represent movement of the cursor towards the target zone.

All participants performed a total of 252 trials of the IT task. These trials were equally divided between 3 conditions, with each condition consisting of 3 blocks of 28 trials. The entire experiment lasted approximately 60 minutes (each block was ~ 5 minutes, each interspaced by ~ 2 minutes of break time). In one condition the IT stimulus was presented alone with no movement, in a second condition the IT figure was presented at peak-limb velocity, and in a final condition the IT figure was presented during the RT foreperiod. Order of presentation of the experimental conditions was counterbalanced across participants. Prior to the beginning of each condition, a total of 10 practice trials were performed to ensure familiarity with the task. A chronological schematic representation of stimulus display events for each condition is presented in Fig 2.

#### No movement condition (NM)

Participants began by placing their right arm at the home position. A warning tone (200 Hz, 80 dB, 100 ms) was presented, which was followed by an acoustic signal (1000 Hz, 80 dB, 40 ms) which acted as the imperative go-signal in the other conditions. The time interval between the warning and go-signal (foreperiod) varied from 3000 to 3500 ms. A visual fixation cross was presented on the monitor in the location noted above and appeared at the beginning of every trial up until the presentation of the acoustic go-signal. Subsequent to the go-signal, a blank target area was shown for 300 ms. The pi figure was then presented for either 15, 30, 45, 60, 75, 90, or 105 ms, and then backward masked for 400 ms. Pi figure presentation duration was randomized and each duration occurred 4 times within each block. After 400 ms, participants were required to verbally state whether the right or left leg of the pi figure was longest, and how confident they were in their response on a scale from 1 to 5 (1 = not confident at all; 5 = very confident). Once the participant provided their responses, the researcher took note of the answers by entering them into the data collection program and waited approximately 5 seconds before beginning the next trial.

#### Movement with IT paradigm occurring at peak-limb velocity (PV)

Participants began by moving their limb to the home position. Once at the home position, a warning tone (200 Hz, 100 ms) was presented, online visual feedback of the limb position was removed, and the visual fixation cross was presented. A random foreperiod of 3000–3500 ms occurred between the warning tone and the imperative go-signal. Specifically, after the warning signal, a random amount of time between 1000 to 1500 ms was followed by 2 s of data collection prior to the fixation cross disappearing and presentation of the acoustic go-signal (1000 Hz, 100 ms). Participants were required to react as quickly as possible to the go-signal by making a 30 deg movement to the location of the fixation cross as rapidly as possible. Although the fixation cross was located 30 degrees from the target, a movement was considered accurate if it ended between the two vertical lines resulting in a target width window of approximately 10 deg. Data from the potentiometer, which was sampled at 4000 Hz (National Instruments, PCIe-6321), was used online to calculate the point at which the limb reached peak velocity. This was accomplished by performing a point-by-point differentiation of a mean of the previous 10 ms of displacement data. We then used a custom written peak-detection algorithm to determine when a peak occurred in the velocity data that was in excess of 200 deg/s. Testing of this procedure found that true peak velocity was typically within 5 ms of the point identified by the online algorithm. Once peak velocity was reached, the pi figure was immediately presented inside the target region at the location of the fixation cross for between 15 and 105 ms (in 15 ms steps) and was then backward masked for 400 ms. Directly following mask offset, terminal feedback of hand position was provided (i.e., the red dot reappeared) and subjects verbally stated which leg of the pi figure was longest as well as their confidence in the choice. Participants then moved back to the home position for the start of the next trial.

#### Movement with IT paradigm occurring during foreperiod (FP)

Participants began by moving the limb to the home position. The warning tone was presented and online visual feedback of the limb position was removed. The visual fixation cross was visible for between 1000–2200 ms after the warning signal. Subsequent to the disappearance of the fixation cross, the target area remained blank for 300 ms, which was followed by the pi figure being presented for between 15 and 105 ms (in 15 ms steps), and then backward masked for 400 ms. The target area was again blanked for a further 0–1785 ms to complete the remainder of the 3000–3500 ms foreperiod. The go-signal was then presented and participants were required to move to the location of the fixation cross as rapidly as possible. Terminal feedback of hand position was provided at the end of every trial. Following their movement, participants verbally stated which leg they thought was longest and with their confidence score. They then moved their arm back to the home position to begin the next trial.

#### Data reduction and analysis

Timing of experimental parameters, display of stimuli and feedback, and collection of data was controlled using a custom-built LabVIEW program (National Instruments Inc.). The potentiometer signal was sampled at 4000 Hz for 4 s starting 2 s prior to the go-signal (NM and PV conditions) or 715–2490 ms prior to the go-signal (FP condition). For the IT task, verbal responses congruent with the visual stimulus were classified as correct. Proportions of correct responses were determined for all participants for the three conditions in each Experiment at all visual stimulus durations. The proportional data were then transformed using an Aligned Rank Transform (ART), a statistical method used for non-parametric factorial repeated measures ANOVAs [9], and analyzed using a 3 movement condition (Experiment 1: NM, FP, PV; Experiment 2: NM, PV, PV-FV) x 7 display duration (15, 30, 45, 60, 75, 90, 105 ms) repeated measures ANOVA. The locus of any significant main effects was determined by using pairwise comparisons. Additionally, a Chi-square analysis at each visual stimulus duration was performed for all three conditions to determine the time at which the proportion of correct responses was significantly greater than chance.

For each participant, mean self-reported confidence in the IT responses was calculated for each movement condition and IT display duration. The ART method was then used to transform the self-reported confidence means, which were then analyzed using a 3 movement condition x 7 display duration RM ANOVA. Multiple Wilcoxon signed rank tests were performed to determine the locus of any interaction effects.

For the reaching movements, movement onset was determined as the first point in time where displacement was greater than 0.2 deg from the home position. Peak velocity and peak displacement were defined as the velocity and displacement values corresponding to when angular acceleration crossed zero the first and second times, respectively, after movement onset. Movement final position was defined as the first point at which angular velocity fell below, and remained below, 8 deg/s for at least 150 ms. Kinematic features of the movement, including movement onset time, time to peak velocity, peak velocity, time to peak displacement, peak displacement, final displacement, and movement time, were compared between the FP and PV conditions using a 2 movement condition x 7 display duration RM AVOVAs. Post-hoc analyses were performed where needed using Tukey’s HSD multiple comparisons tests. All analyses were performed using SPSS 21 statistical software package for Windows (IBM Inc., Armonk, NY, USA), and alpha was set *a priori* to *p* ≤ .05 for all tests.

### Results

#### Proportion of correct responses

**Fig 3:**
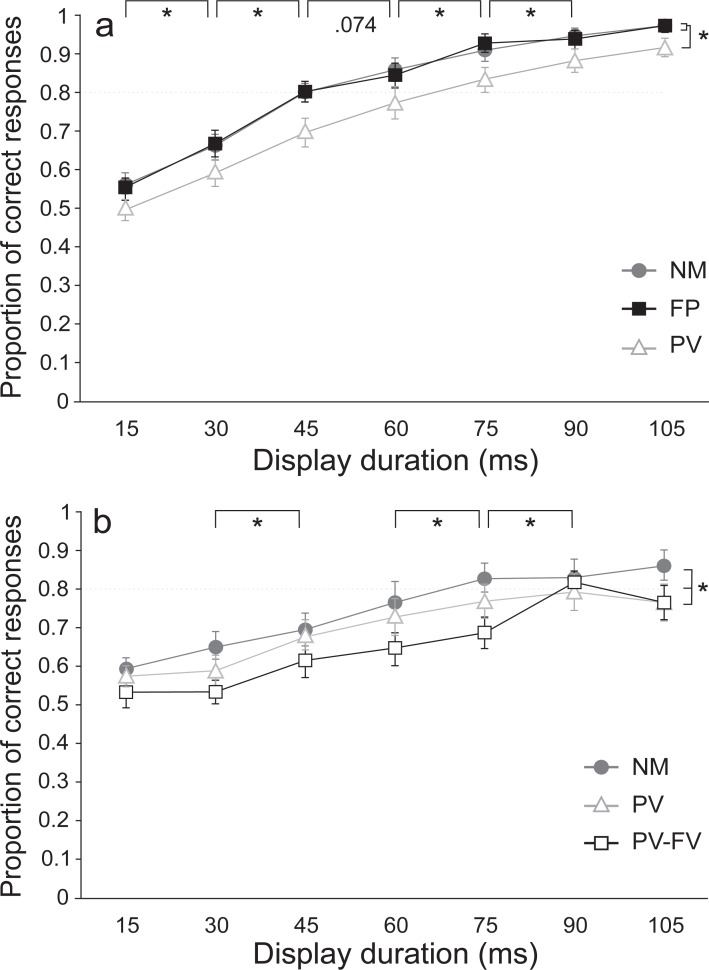
**Inspection time (IT) task performance as a display duration (ms) in Experiment 1 (panel A) and Experiment 2 (panel B).** Panel A: mean (SE) proportion of correct identifications of the longest leg of the IT stimulus in Experiment 1. Movement conditions include: no movement (NM; grey circles), foreperiod (FP; black squares), and peak velocity (PV; white triangles). Note that although the proportion of correct responses increased with display duration for all movement conditions, a significantly (*) lower proportion of correct responses was seen in the PV condition at all IT stimulus durations compared to the NM and FP conditions. Panel B: mean (SE) proportion of correct identifications for the three different movement conditions in Experiment 2. Movement conditions include: no movement (NM; grey circles), full vision (PV-FV; white squares), and peak velocity (PV; grey triangles). Note that while the proportion of correct responses generally increased with display duration, it was significantly (*) different between movement conditions, with the lowest proportion of correct responses in the PV-FV and the highest in the NM condition.

The proportion of correct responses observed in each condition and at each time are shown in Fig 3A. Statistical analysis of the proportion of correct responses confirmed significant main effects for both movement condition [F(2,54) = 10.099, *p* < .001, n^2^_p_ = .272] and display duration [F(6,162) = 100.491, *p* < .001, n^2^_p_ = .788 ], with no significant interaction between the factors (*p* = .454). Post-hoc pairwise comparisons between conditions showed that the percentage of correct responses was significantly lower in the PV condition compared to both the NM (*p* = .002) and FP (*p* = .006) conditions. However, the NM and FP conditions were not significantly different from each other (*p* = 1.000). For the main effect of time, the percentage of correct responses at each successively longer display duration was significantly different from the previous display duration, except at the durations of 45 and 60 ms and at durations of 90 and 105 ms, which were not significantly different (all *p*-values > .05). Chi-square analysis showed that when the pi figure was displayed for 30 ms the proportion of correct responses was significantly greater than chance in the NM (χ^2^ = 9.85, *p* < .05) and FP (χ^2^ = 11.07, *p* < .05) conditions. However, the proportion of correct responses in the PV condition only became significantly greater than chance at the 45 ms duration (χ^2^ = 15.23, *p* < .05). Finally, mean performance in the IT task reached 80% [10] at the 60 ms duration in both the NM and FP conditions, but required 75 ms in the PV condition to surpass this threshold (Fig 3A).

#### Confidence ratings

**Fig 4:**
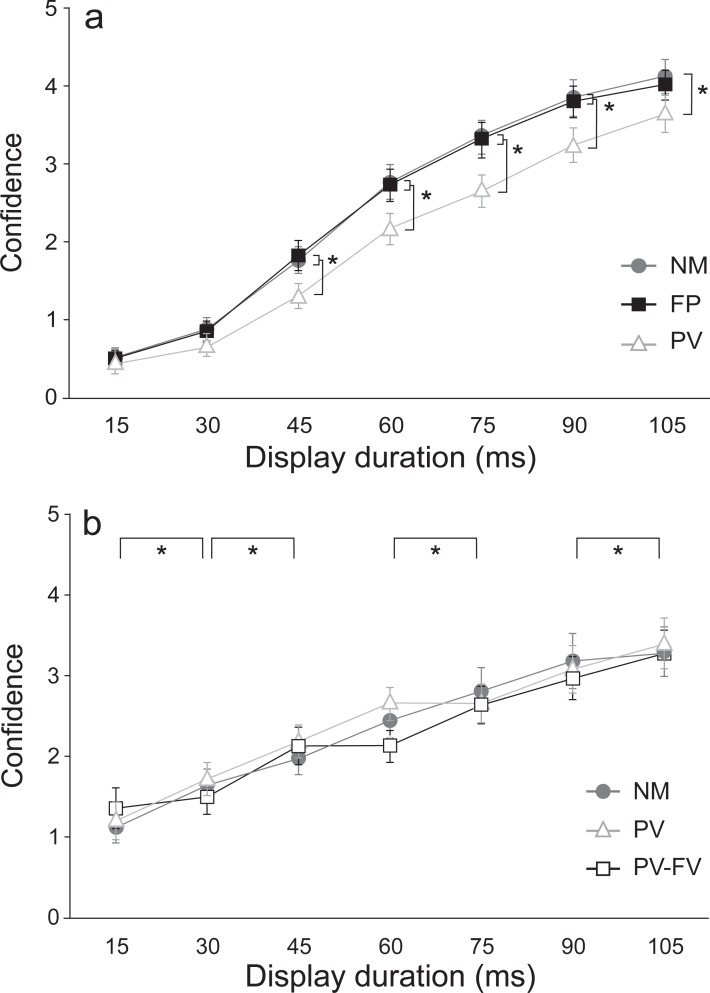
Mean (SE) confidence ratings in the response provided to the IT stimulus. Ratings (1 = not confident at all; 5 = very confident) are shown as a function of stimulus presentation duration in Experiment 1 (panel A) and Experiment 2 (panel B). Panel A: Experiment 1 movement conditions include: no movement (NM; grey circles), foreperiod (FP; black squares), and peak velocity (PV; white triangles). Panel B: Experiment 2 movement conditions include: no movement (NM; grey circles), full vision (PV-FV; white squares), and peak velocity (PV; grey triangles). * signifies significant differences (*p* < .05).

Confidence ratings for responses made in each condition and at each stimulus duration are shown in Fig 4A. Analysis of confidence ratings showed main effects for both condition [F(2,54) = 13.628, *p* < .001, n^2^_p_ = .335] and display duration [F(6,162) = 190.552, *p* < .001, n^2^_p_ = .876]. However, these were superseded by a significant interaction effect between condition and duration [F(12,324) = 3.335, *p* = .004, n^2^_p_ = .110]. Wilcoxon signed rank post-hoc tests showed that within each condition, confidence increased with each increase in duration (all *p*-values < .008). In addition, Wilcoxon signed rank post-hoc tests performed at each display duration showed that confidence ratings were significantly higher in the NM as compared to the PV condition at all display durations ≥ 45 ms (all *p*-values < .016), although they were not different at display durations of 15 or 30 ms (*p*-values > .05). Similarly, confidence ratings were significantly higher in the FP as compared to the PV condition at display durations between 45 to 90 ms. Lastly, NM and FP conditions were not significantly different from each other at any of the display durations (all *p*-values >.05).

#### Movement kinematics

**Fig 5:**
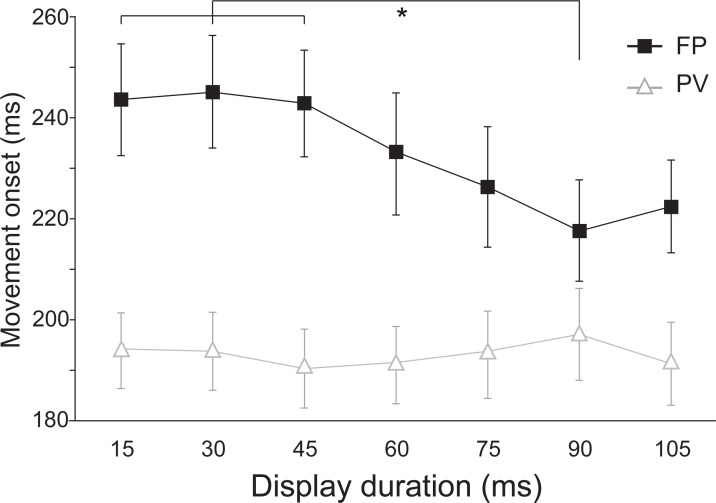
Mean (SE) movement onset time (ms) as a function of stimulus presentation duration (ms). Black squares show data from the foreperiod (FP) condition, and white triangles show data from the peak velocity (PV) condition. * signifies significant differences (*p* < .05).

Significant differences were found in movement kinematics between the two conditions involving a targeted limb movement (FP versus PV). Specifically, analysis of movement onset (i.e., reaction time) showed a significant main effect for both condition [F(1,27) = 31.270, *p* < .001, n^2^_p_ = .537] and display duration [F(6,162) = 2.213, *p* = .044, n^2^_p_ = .076], but these were superseded by a significant interaction effect between the factors [F(6,162) = 2.885, *p* = .011, n^2^_p_ = .097] (Fig 5). A Tukey’s test for multiple comparisons confirmed that RT was significantly longer at all display durations in the FP condition as compared to the PV condition (all *p*-values < .05). In addition, while RT was not different across display durations in the PV condition, RT appeared to decrease as display duration increased in the FP condition, and was significantly shorter at the 90 ms duration compared to the 15, 30, and 45 ms durations (for details see Fig 5).

While there were no significant main effects or interaction observed for peak velocity (all *p*-values > .05), all other kinematic variables revealed a significant main effect of movement condition (see Table 1). Analysis confirmed that the FP condition exhibited a longer time to reach peak velocity [F(1,27) = 6.836, *p* = .014 , n^2^_p_ = .202], larger peak displacement [F(1,27) = 6.178, *p* = .019, n^2^_p_ = .186], as well as a longer time to peak displacement [F(1,27) = 10.535, *p* = .003, n^2^_p_ = .281] compared to the PV condition. In addition, larger final displacement was observed in the FP condition [F(1,27) = 5.214, *p* = .030, n^2^_p_ = .162], as well as a longer time to final displacement (i.e., movement time) [F(1,27) = 9.411, *p* = .005, n^2^_p_ = .258], compared to the PV condition. The main effect of display duration and movement condition by display interaction were not significant for any of the variables reported (all *p*-values > .05).

**Table 1:** Mean (SE) values for kinematic variables for the foreperiod (FP) and peak velocity (PV) conditions in Experiment 1.

	FP		PV
Peak Velocity (deg/s)	272.6 (15.1)		280.2 (14.2)
Time to Peak Velocity (ms)	202.2 (9.3)	*	187.5 (8.7)
Peak Displacement (deg)	36.2 (1.4)	*	33.0 (1.0)
Time to Peak Displacement (ms)	413.4 (22.4)	*	376.8 (19.7)
Final Displacement (deg)	34.2 (1.3)	*	31.3 (0.9)
Movement time (ms)	491.2 (24.7)	*	448.2 (21.4)

* indicates significant main effect of condition.

### Discussion

The purpose of the first Experiment was to determine if visual processing speed was enhanced when either preparing to move the limb or when the limb was moving at peak velocity compared to a no-movement condition. Unexpectedly, results showed that presenting the IT stimulus at peak-limb velocity significantly *degraded* performance in the IT task at all stimulus durations (Fig 3A). A possible explanation for this result is that removing visual feedback during the movement led to participants down-regulating vision. Specifically, it might be argued that visual upregulation would not be expected in a situation where no visual feedback was available and could not be used to refine the movement. In order to address this issue a second experiment was conducted where online visual position feedback was provided during movement production.

## Experiment 2

### Material and methods

#### Participants

Eighteen healthy individuals (12 F, 6 M), with a mean age of 24.1 years (SD = 5.5) participated in Experiment 2. Two participants participated in both experiments. All participants self-reported as right-handed or ambidextrous.

#### Procedure and data analysis

The methods used in Experiment 2 were identical to those used in Experiment 1 except for the following: In Experiment 2 the FP condition was replaced by a second PV condition in which a visual representation of limb position was provided throughout the movement. Thus, participants completed the NM and PV conditions from Experiment 1 and a full vision (PV-FV) condition in which the IT paradigm also occurred at peak-limb velocity and the position of the limb was provided throughout the trial via a small red circle (0.5 cm) that corresponded directly to the position of the manipulandum handle. Each trial started with participants moving their limb such that the red dot would be located at the home position. A visual fixation cross was then presented in the location of the movement goal at the beginning of every trial up until the presentation of the go signal. Participants were required to fixate the cross during the trial, despite the provision of visual feedback. The warning tone was presented, and after a random foreperiod of 3000 to 3500 ms the acoustic go-signal was presented. Participants moved as rapidly as possible to the location where the fixation cross had been (between the two vertical lines on screen). Following movement completion and once the mask had been removed, subjects verbally stated which leg of the pi figure was longest as well as their confidence in the choice. Participants then moved back to the home position for the start of the next trial. Data collection, data reduction, and analyses were conducted like in Experiment 1, except that the foreperiod (FP) condition was replaced by the PV-FV condition.

### Results

#### Proportion of correct responses

For Experiment 2, the proportion of correct responses observed in each condition and at each time are shown in Fig 3B. Statistical analysis of the proportion of correct responses confirmed significant main effects for both movement condition [F(2,34) = 6.801, *p* = .008, n^2^_p_ = .808] and display duration [F(6,102) = 24.189, *p* < .001, n^2^_p_ = .587], but no significant interaction between the factors (*p* = .619). Similar to Experiment 1, post-hoc pairwise comparisons between movement conditions showed that the percentage of correct responses was significantly lower in the PV condition compared to the NM condition (*p* = .032). Additionally, the PV-FV condition showed a significantly lower percentage of correct responses compared to both the NM condition (*p* = .009) as well as the PV condition (*p* = .050). For the main effect of time, the percentage of correct responses at each successively longer display duration was significantly different from the previous display duration, except between durations of 15 and 30 ms, durations of 45 and 60 ms, and durations 90 and 105 ms, which were not significantly different (*p*-values > .05). Chi-square analysis showed that in the NM condition the proportion of correct responses was significantly greater than chance when the pi figure was displayed for 60 ms (χ^2^ = 5.19, *p* < .05). However, proportion of correct responses only became greater than chance at 75 ms in the PV condition (χ^2^ = 5.19, *p* < .05) and at 90 ms in the PV-FV condition (χ^2^ = 7.13, *p* < .05). Finally, the duration at which an 80% criterion was reached was 75 ms in the NM conditions, 90 ms in the PV-FV condition, and did not exceed 80% at any IT display duration in the PV condition (Fig 3B).

#### Confidence ratings

In Experiment 2, confidence ratings for responses made in each condition and at each stimulus duration are shown in Fig 4B. Analysis of confidence ratings showed no main effect for movement condition (*p* = .725) but a significant main effect of display duration [F(6,102) = 10.691, *p* = .002, n^2^_p_ = .386]. There was also no significant interaction effect between movement condition and duration (*p* = .184). Post hoc analysis showed that confidence in the given response significantly increased with each increasing display duration except between 45 and 60 ms, and between 75 and 90 ms, which were not significantly different (*p*-values > .05).

#### Movement kinematics

For Experiment 2, no significant differences between the two conditions involving targeted limb movements (PV-FV versus PV) were found for any of the movement kinematic variables (all *p*-values > .25), while reasonable target accuracy was maintained (PV-FV = 3.0 deg target overshoot, PV = 2.9 deg target overshoot).

### Discussion

The purpose of the second experiment was to determine whether provision of online visual feedback during action was required for upregulation of visual processing. In contrast to our expectations, providing online visual feedback led to an even larger detriment in performance of the IT task in comparison to when no online visual feedback was available. Given these results, the degradation in visual processing (i.e., an increase in the time required to process the same amount of information) observed in the first experiment cannot be explained by a lack of online visual feedback.

## General discussion

The present study aimed to establish if inspection time is altered by preparation and/or execution of a goal-directed movement. Inspection time has been suggested to provide insight into processing time for visual perception [8, 10]. To test visual processing, a perceptual IT task was completed prior to and during a goal-directed limb movement. We expected that if visual processing is upregulated at peak-limb velocity then presentation of the IT stimulus during the fastest part of the movement would result in improved IT performance in comparison to that observed in the NM condition. Likewise, if visual perceptual processing was enhanced by motor preparation, then performance in the IT paradigm would be better when the IT stimulus was presented during the RT foreperiod of a goal-directed limb movement in comparison to when it was presented while sitting idly (NM condition). These expectations were based on previous literature that found improved visual perceptual ability when visual stimuli were presented during a RT foreperiod [2] and at peak-limb velocity [3]. Contrary to expectations, however, the first experiment showed that performance on the IT task in the FP condition was not significantly different from performance in the NM condition, providing no evidence for visuo-perceptual enhancement due to motor preparation. In the PV condition, performance on the IT task was significantly poorer in comparison to the NM condition, even though the stimuli were presented at similar times following the acoustic stimulus (Fig 3A). Moreover, the second experiment replicated and confirmed these results, and showed further degradation in IT performance when online visual feedback was provided (Fig 3B).

One possible explanation for the decreased performance seen in the IT task when it was associated with movement may simply be reflective of the allocation of limited attentional resources. That is, it is possible that participants used selective attention to filter and process visual information that was essential to the motor task [11] and thus actively inhibited non-target-related information (the IT stimulus) [12]. Similarly, the indirect nature of the feedback provided (cursor-based versus direct feedback) may have led to an increase in attentional requirements, although further study is required to investigate this possibility. Alternatively, it is possible that participants prioritized the movement task over the perceptual task by predominantly attending to task-relevant visual stimuli [13]. This “attentional resources” explanation is further supported by the results of Experiment 2, which showed not only a degraded IT task performance when the limb was moving at peak velocity compared to not moving, but also showed a further degradation in IT performance when additional visual information (online visual feedback) was available. Indeed, a number of experiments have shown degraded secondary task performance when the primary task involves manipulandum-based movement execution. For example, Bratzke and colleagues [14] used a psychological refractory period paradigm involving a ballistic left arm movement, followed by a right-hand key-press task and found performance decrements in the second task, which they attributed to execution-related interference. Similarly, Maslovat and colleagues [15] showed that performance of a continuous manual tracking task reduced the attentional resources available to perform a secondary wrist-extension RT task. Although both these previous experiments involved multiple motor-based tasks, a similar effect may have occurred in the current study whereby response execution of the movement task interfered with the processing of other responses.

A second possible explanation for the poorer performance in the IT task during the movement related conditions compared to the NM condition involves the presentation location of the visual stimulus to be discriminated. In the two experiments presented here, the visual stimulus was presented in focal vision. In contrast, in the previous study by Tremblay and Nguyen [3], visual stimuli were presented six cm below the fixated reach target (i.e., in peripheral vision). Given this, it is possible that focal and peripheral vision may be processed and weighted differently during a goal directed movement, such that peripheral vision is enhanced at peak-limb velocity, while focal vision remains unaffected, or indeed degraded. In support of this proposal, Cao and Haendel [16] recently showed an enhancement in visual processing of peripheral information during locomotion, a phenomenon hypothesized to occur due to the importance of peripheral navigational cues during this type of action [17]. This purported enhancement in peripheral visual processing exerted suppressive effects on central visual processing (i.e., central vision was degraded due to the increased peripheral input).

It is possible, then, that a simple goal-directed movement may induce the same effect; that is, enhance peripheral visual processing by shifting attention to the periphery, which in turn may degrade processing of visual information that falls on the fovea [16]. An important note to consider is that the suppressive effects observed by Cao and Haendel [16] were only present during locomotion, and disappeared when participants stood still. Similarly, in the present study, the IT performance was only degraded during movement (PV condition), and was similar when performed before versus without movement (FP/NM, respectively). In the absence of movement, attention could still be focused on central vision, without suppression. In support of focal vision degradation during movement, we found decreased accuracy in the IT task and decreased confidence ratings associated with the IT stimulus in the movement conditions compared to the non-movement condition. That is, in comparison to the NM condition, confidence ratings were consistently lower at most of the display durations in the PV condition (Fig 4A). Given that confidence ratings have been proposed to be directly proportional to discrimination ability and accuracy [18], the lower confidence ratings further suggest diminished perceptual processing of the visual stimulus in the movement conditions in the current experiment. It is certainly plausible that in contrast to the perceptual processing degradation shown here, different effects on visual processing may have been observed if the IT stimulus had been presented in peripheral, as opposed to central vision. In light of Goodale and Milner’s dual streams model [1], the dorsal and ventral streams play a large role in the *detection*, and *identification* of visual stimuli, respectively. As such, the ventral stream is thought to be susceptible to visual illusions, while the dorsal stream is thought to resist such illusory effects. Given this, it is unclear as to whether the increased resistance to the audio-visual illusion observed in Tremblay and Nguyen’s [3] study was due to the increased dorsal stream processing (enhanced visual detection) or to increased ventral steam processing (decreased susceptibility to illusion).

The results of the work by Tremblay and Nguyen [3] may be due to enhanced visual processing of stimuli in the periphery. Alternatively, the increased resistance to the audio-visual fusion illusion they found when the limb was moving at peak-limb velocity [3] may have been due to improved multisensory processing at peak-limb velocity. Indeed, these authors suggested a similar possibility with the limb movement resulting in an increased signal-to-noise ratio in the relevant sensory channels. This suggestion is also supported by a study showing that in the absence of auditory cues, performing a fast limb movement degraded the ability to detect the number of visual flashes presented [5]. While the present results cannot speak to this possibility, they do indicate that the previous results can likely not be attributed to enhanced visual perceptual processing alone.

A secondary finding of Experiment 1 is that no change in IT performance was observed when it was presupposed that participants would be concurrently preparing the limb movement. This contrasts with results provided by Hagura et al. [2], who showed a significant increase in visual letter identification when visual stimuli were presented 300 ms before the go-signal in a RT task. One explanation for the discrepancy in results between paradigms may be related to when the IT stimulus was presented during the foreperiod (FP) condition in Experiment 1. Specifically, in the FP condition, the IT stimulus was presented between 400 and ~2200 ms before the go-signal. In the study by Hagura et al. [2] only letters that were presented < 300 ms before the go-signal were more likely to be identified correctly. In contrast, and similar to our findings, letters that were presented greater than 300 ms prior to the go-signal were identified with a similar level of accuracy regardless of whether or not participants were planning to move. However, one major caveat in the current study is that the level of movement preparation prior to, or during the IT task in the FP condition was not probed. Because the go-signal could occur immediately following offset of the mask, it is likely that participants engaged in some level of preparatory processing prior to / during the IT task. Indeed, mean movement onset times were below 250 ms for all IT durations in the FP condition (Fig 5), suggesting advanced preparation. Assuming preparatory processing *was* occurring during the IT task in the FP condition, the similarity of results at all display durations compared to the NM condition, indicates that response preparation did not lead to improved visual perception. However, given that we cannot say with certainty how much preparation had occurred prior to the IT stimulus, and that participants still had ~900 ms on average to prepare for the motor action after the end of the IT task, it is very possible that the bulk of motor preparation could have taken place following mask offset in the FP condition. In this case, it would not be surprising that no differences were observed between the NM and FP conditions in terms of IT task accuracy, as the small amount of motor preparatory activity would have little effect on visual discrimination. Together these results suggest that if motor preparation does enhance visual processing, this effect may only occur during a narrow window of time prior to response initiation, which may account for lack of visuo-perceptual enhancement observed during the RT foreperiod of Experiment 1.

Finally, movement kinematics in both experiments were compared between conditions in which movements were completed. Of particular note, results from Experiment 1 showed that RT was significantly longer when the IT task was presented during the RT foreperiod (i.e., the FP condition) compared to when it was presented during movement (PV condition) (Fig 5). Moreover, this RT difference was greater at shorter IT durations compared to longer ones. That is, when the perceptual task was more difficult (e.g., the IT stimulus displayed for 15 or 30 ms), RT was significantly longer, while when the perceptual task was easier (e.g., display durations of 90 or 105 ms), RT was shorter. In addition, movements were generally slower in the FP condition in comparison to the PV condition at all IT durations (Table 1). These results suggest that engagement in a perceptually challenging task may interfere with motor preparation prior to an imperative go-stimulus and this interference may also affect movement production. A previous study showed similar results when participants were required to engage in a cognitive task with two levels of difficulty while preparing to make a quick limb movement in a RT task following a control tone or a startling acoustic stimulus (SAS) [19]. Findings showed that when the cognitive task was more difficult, RT increased significantly in comparison to when it was easier or when the motor task was performed alone. Moreover, given that RTs observed as a result of involuntary response triggering due to startle [20] were also longer during the performance of a cognitive task, it was concluded that preparatory activation level for the secondary motor task was also affected by the cognitive requirements. It is possible that a difficult cognitive task (e.g., IT stimulus displayed for 15 or 30 ms) may have challenged the participants’ ability to maintain focus or alertness until the presentation of an impending target, which has been shown to significantly increase RT [21]. Moreover, given that “peak readiness” cannot be maintained for long periods of time (~300–400 ms) [22–24], it is possible that the combination of a challenging cognitive task and having to maintain preparedness for a long period of time before the presentation of an impending target could have decreased the level of alertness and resulted in an overall slower RT.

Together with these previous findings, the current RT results (Fig 5) along with the slower, less accurate movements (Table 1) following performance of the IT task during the RT foreperiod suggest that engaging in a perceptually demanding task may have interfered with preparation of the targeted movement.

## Conclusion

In summary, no evidence for increased visual perceptual processing at peak velocity of a goal-directed movement was found in the present experiments. In contrast, the speed of visual processing appears to have decreased during movement production, which was further exacerbated by the provision of online visual feedback. The most likely explanation for these results is that during movement production fewer attentional resources were available to perform the IT task concurrently with the movement task. Although no improvement in visual inspection time was found during the foreperiod of a goal-directed movement, these findings may be attributable to the presentation time of the visual stimuli with respect to the go-signal. However, the present results do suggest that the requirement to perform a perceptually demanding task interferes with the preparation of an upcoming movement. Overall, these results indicate that the perceptual processing of visual information is modulated based on perceived task-related priorities during movement execution.
